# Tissue Cytokine IL-33 Modulates the Cytotoxic CD8 T Lymphocyte Activity During Nutrient Deprivation by Regulation of Lineage-Specific Differentiation Programs

**DOI:** 10.3389/fimmu.2019.01698

**Published:** 2019-07-24

**Authors:** Caroline Dreis, Florian M. Ottenlinger, Mateusz Putyrski, Andreas Ernst, Meik Huhn, Katrin G. Schmidt, Josef M. Pfeilschifter, Heinfried H. Radeke

**Affiliations:** ^1^pharmazentrum Frankfurt/ZAFES, Institute of Pharmacology and Toxicology, Hospital of the Goethe University, Frankfurt am Main, Germany; ^2^Project Group Translational Medicine and Pharmacology TMP, Fraunhofer Institute for Molecular Biology and Applied Ecology IME, Frankfurt am Main, Germany; ^3^Institute of Clinical Pharmacology, Goethe-University, Frankfurt am Main, Germany

**Keywords:** IL-33, bioactivity, CD8^+^ T lymphocytes, ST2L, nutrient deprivation, mTOR

## Abstract

IL-1 family member IL-33 exerts a variety of immune activating and regulating properties and has recently been proposed as a prognostic biomarker for cancer diseases, although its precise role in tumor immunity is unclear. Here we analyzed *in vitro* conditions influencing the function of IL-33 as an alarmin and a co-factor for the activity of cytotoxic CD8^+^ T cells in order to explain the widely discussed promiscuous behavior of IL-33 *in vivo*. Circulating IL-33 detected in the serum of healthy human volunteers was biologically inactive. Additionally, bioactivity of exogenous recombinant IL-33 was significantly reduced in plasma, suggesting local effects of IL-33, and inactivation in blood. Limited availability of nutrients in tissue causes necrosis and thus favors release of IL-33, which—as described before—leads to a locally high expression of the cytokine. The harsh conditions however influence T cell fitness and their responsiveness to stimuli. Nutrient deprivation and pharmacological inhibition of mTOR mediated a distinctive phenotype characterized by expression of IL-33 receptor ST2L on isolated CD8^+^ T cells, downregulation of CD8, a transitional CD45RA^low^RO^low^ phenotype and high expression of secondary lymphoid organ chemokine receptor CCR7. Under nutrient deprivation, IL-33 inhibited an IL-12 induced increase in granzyme B protein expression and increased expression of *GATA3* and *FOXP3* mRNA. IL-33 enhanced the TCR-dependent activation of CD8^+^ T cells and co-stimulated the IL-12/TCR-dependent expression of IFNγ. Respectively, *GATA3* and *FOXP3* mRNA were not regulated during TCR-dependent activation. TCR-dependent stimulation of PBMC, but not LPS, initiated mRNA expression of soluble IL-33 decoy receptor sST2, a control mechanism limiting IL-33 bioactivity to avoid uncontrolled inflammation. Our findings contribute to the understanding of the compartment-specific activity of IL-33. Furthermore, we newly describe conditions, which promote an IL-33-dependent induction of pro- or anti-inflammatory activity in CD8^+^ T cells during nutrient deprivation.

## Introduction

Interleukin (IL)-1 family member IL-33 (IL-1F11) was recently identified as the missing ligand for ST2L (IL-1RL1, now IL-1R4) and was primarily associated to immune cells with a T helper 2 (Th2) phenotype (1). IL-33 exerts a dual function as an intra-nuclear negative regulator of NF-κB and extracellularly as a cytokine potently co-stimulating adaptive immune responses far beyond Th2 immunity, thus offering new targets for the activation of anti-tumoral cytotoxic T cells (2–4). IL-33 detected by immunogenic techniques in serum is used as a prognostic biomarker for chronic heart failure and multiple sclerosis, but has recently also been proposed as a prognostic marker for cancer diseases (5–8). The role of IL-33 in cancer immunity however remains controversial. The first reason is that information on the compartment-specific regulation of IL-33 bioactivity is missing. Bioactivity of extracellular IL-33, which is passively released upon necrosis or tissue damage is highly regulated by extensive conformational changes of the receptor binding IL-1-like cytokine domain, resulting in stabilization or complete loss of receptor binding (9–11). A multitude of mechanisms is known to inhibit IL-33 bioactivity in blood, suggesting complete systemic inactivity of IL-33. Secondly, IL-33 acts as a broadly active, and variable co-stimulator for feedback loops enhancing T cell differentiation programs for Th1, Th2, or Treg fates (4). We and others have previously provided evidence implicating a major involvement of IL-33 in Th1 immunity and cytotoxic CD8^+^ T cells. IL-33 and IL-12 co-induced T cell receptor (TCR) triggered secretion of Interferon-γ (IFNγ) in murine CD8^+^ T cells (12–14). Furthermore, IL-33 inhibits tumor growth by contributing to the proliferation, activation and infiltration of CD8^+^ T cells and NK cells (15, 16). Co-factors supporting the induction of either differentiation pathway by IL-33 are yet to be identified. Additionally, impairment of nutrient delivery resulting from an abnormally structured vascular system in tumor tissue induces diverse cellular stress responses and influences the fitness of tumor infiltrating T cells (17, 18). Nutrient availability is however fundamental for cell growth and survival. Mammalian target of rapamycin (mTOR) is an atypical serine/threonine protein kinase which orchestrates energy-consuming anabolic and energy-producing catabolic pathways. mTOR is activated under nutrient rich conditions, inhibited during nutrient deficiencies and has a central role in the regulation of autophagy, T cell differentiation and memory generation (19, 20). In a cancer model mimicking the tumor microenvironment, IL-33 directly affected the survival of tumor cells by activation of mTOR (21). Our study focused on the regulation of IL-33 bioactivity by soluble decoy receptor ST2 (sST2), by intra-molecular modifications and on the cellular level by differentially modulating IL-33 responsivity under nutrient deprivation. Here we will demonstrate that IL-33 lacks bioactivity in blood, provoking a new focus on the role of IL-33 in tissue. Importantly, we show for the first time, that nutrient deprivation induces ST2L expression on CD8^+^ T cells. Those cells can be modulated in an IL-33 dependent fashion to generate regulatory or effector T cells, implicating new findings with relevance for tumor immune therapy.

## Materials and Methods

### Materials

Interleukins IL-33, IL-12p70 (further named IL-12) and IL-1β were purchased from PeproTech (Hamburg, Germany) and diluted in PBS/0.1% BSA. IL-33 oxidation mutant C208S/C232S and its wild type counterpart sensitive to oxidation were obtained from AdipoGen (San Diego, USA). Cell culture plates and flasks were obtained from Greiner bio-one (Frickenhausen, Germany). All cell culture reagents were purchased at the highest purity and cell culture grade. Supplements for HEK293-ST2L or T cell medium were bought from Life Technologies (Darmstadt, Germany) and Invitrogen (NY, USA) if not stated otherwise. Concentrations of IL-1β and IL-33 in serum or plasma or IFNγ in culture supernatants were quantified with ELISA kits (R&D, Minneapolis, USA) according to the manufacturer's instructions.

### Generation of Recombinant IL-33 Isoforms

IL-33 isoforms were generated from the full-length human IL-33 expression plasmid pMyc-hIL-33-preFl (kindly provided and designed by Prof. Michael U. Martin, Justus-Liebig University Giessen, Germany). cDNA of the IL-33 isoforms were generated from pMyc-hIL-33-preFl according to the predicted cleavage sites of cathepsin G or chymase producing aa_95−270_ or caspase-3/-7 producing aa_179−270_. For expression of the isoforms in *Escherichia coli*, the cDNAs were subcloned into expression vector pET28(+)-Ub. IL-33 isoforms were purified according to the method described in Mora et al. (22) with a column buffer composed of 50 mM phosphate pH 7.5, 600 mM NaCl, 4% v/v glycerol and 1 mM β-mercaptoethanol. His-Tag was removed by cleavage with thrombin and proteins were further purified by gel filtration (FPLC, GE Healthcare). Endotoxin levels were <0.01 endotoxin units/μg of protein.

### Cultivation and Stimulation of HEK293-ST2L Reporter Cells

HEK-Blue IL-33/IL-1β (HEK293-ST2L) cells were purchased from Invivogen (Toulouse, France) and cultivated according to the manufacturer's protocol in DMEM (Greiner bio-one, Frickenhausen, Germany) with 5% FCS (GE Healthcare, IL, USA), 2 mM l-glutamine, 100 IU/ml penicillin and 100 IU/ml streptomycin at 5% CO2, 37°C. 30 μg/ml blasticidin, 200 μg/ml hygromycin B Gold and 100 μg/ml zeocin were added after the second passage to maintain the plasmids encoding IL1R1, co-receptor IL-1RAcP, ST2L, and the gene for SEAP. To analyze IL-33 or IL-1β bioactivity, 5 × 10^4^ HEK293-ST2L cells were resuspended in 180 μl medium and were co-incubated with 20 μl of sample. In some experiments, 5 × 10^4^ HEK293-ST2L cells were resuspended in 160 μl and co-incubated with 20 μl sample and 20 μl serum or plasma for a total final volume of 200 μl. For inhibition of proteases, protease inhibitor cocktail (Sigma-Aldrich, Steinhein, Germany) was added in a dilution of 1:160 directly to the cell suspension. After a 22 h incubation period, 40 μl of medium supernatant were co-incubated with 160 μl QUANTI-blue substrate (Invivogen, Toulouse, France) and SEAP activity was measured photometrically at 635 nm for 2 h every minute. Cell viability of HEK293-ST2L cells was assessed by MTT assay to exclude cytotoxic effects mediated by the samples (Roche Life Sciences, Basel, Switzerland).

### Serum Samples and Primary Cell Isolation

Serum samples were drawn from healthy male donors during blood donation and stored at −80°C until further preparation. Human PBMC and CD8^+^ T cells were isolated using Ficoll-Histopaque 1,077 gradients (Sigma-Aldrich, Steinheim, Germany). Plasma obtained from density centrifugation was set aside for bioassay experiments.

### Stimulation of Isolated CD8^+^ T Cells and PBMC

CD8^+^ T cells were isolated from PBMC by negative selection using EasySep^TM^ Human CD8^+^ T Cell Isolation Kit (Stemcell Technologies, Vancouver, Canada). CD8^+^ T cells were seeded out in a density of 0.5 × 10^6^/ml and left untreated in in serum-free T cell medium composed of RPMI (Greiner bio-one, Frickenhausen, Germany), 50 μM β-ME, 1% HEPES buffer solution, 100 IU/ml penicillin and 100 IU/ml streptomycin. If indicated, CD8^+^ T cells were cultivated with human AB serum (Sigma-Aldrich, Steinheim, Germany) in a final concentration of 1% (v/v) in T cell medium. For starvation experiments, CD8^+^ T cells were cultivated for 20 h prior further analysis. For inhibition of mTOR, cells were treated with 100 nM of rapamycin (LC Laboratories, MA, USA) in T cell medium containing human serum. Effects of dimethylsulfoxide (DMSO) as diluent for the rapamycin stock were excluded using an appropriate control respecting the final concentration of 0.1% DMSO during stimulation. For TCR-independent stimulation, CD8^+^ T cells were cultivated in serum-free T cell medium for 20 h before addition of 20 ng/ml IL-33, and/or 5 ng/ml IL-12 for 20 h prior analysis. For TCR-dependent stimulation, CD8^+^ T cells were cultivated in serum-free T cell medium, 20 ng/ml IL-33 and/or 5 ng/ml IL-12, and/or 25 μl/ml of ImmunoCult^TM^ Human CD3/CD28/CD2 T Cell Activator (αCD, Stemcell Technologies, Vancouver Canada). PBMC were stimulated in serum-free T cell medium for 20 h with CD3/CD28/CD2 T Cell Activator or 1 μg/ml LPS.

### Analysis of Cell Surface and Intracellular Markers by Flow Cytometry

Expression of cell surface or intracellular markers of CD8^+^ T cell subpopulations were identified by flow cytometry. For CD107a degranulation studies, T cells were plated in serum-free medium in 12-well-plates and cultivated for 20 h prior stimulation. The cells were either left untreated or stimulated with 20 ng/ml IL-33 and/or 5 ng/ml IL-12 and/or 25 μl/ml of αCD T cell activator. Antibodies directed against CD107a (PE, clone H4A3, BioLegend, San Diego, CA, USA) were added at the start of the incubation to detect CD107a translocated to the extracellular membrane during stimulation. After 1 h, monensin (Sigma-Aldrich, Steinhein, Germany), was added in a final concentration of 1 μM. The cells were stimulated for a total of 5 h before harvesting and additional staining of extracellular markers for flow cytometry. For flow cytometry experiments in general, cells were blocked for 15 min at RT with 0.1% PBS/FCS containing human Fc Block (BD Pharmingen, Heidelberg, Germany) prior staining. Cell surface markers were assessed with the following antibodies: CD8-V450 (clone RPA-T8, BD Bioscience, Heidelberg, Germany) T1/ST2L-FITC (clone B4E6, MD Biosciences, Zürich, Switzerland), CD45RA-PE/Cy7 (clone HI100, Biolegend, San Diego, CA, USA), CD45RO-APC (clone UCHL1, Biolegend), CD69-PerCP (clone FN60, Biolegend), CCR7-APC (clone G043H7, Biolegend), or KLRG1-PE (clone 14C2A07, Biolegend). Intracellular staining was performed with antibodies for granzyme B-PE/Cy7 (clone QA16A02, Biolegend), Gata-3-PE (clone TWAJ, Biolegend), FoxP3-Alexa Fluor 647® (clone 259D, Biolegend), T-bet-PE (clone eBio4B10, Invitrogen, NY, USA), or LC3B (ab51520, Abcam, Cambridge, UK) and secondary antibody donkey anti-rabbit IgG Alexa Fluor 488 (Invitrogen, NY, USA) using the FoxP3 Staining Buffer Set (Miltenyi Biotec, Bergisch Gladbach, Germany) according to the manufacturer's protocol. All data were acquired on a FACS Canto II (BD, Heidelberg, Germany). FlowJo (TreeStar, Inc. Ashland, OR, USA) was used for data analysis.

### RNA Isolation, Synthesis of cDNA, and Quantitative Real-Time PCR

CD8^+^ T cells or PBMC were pelleted, and total RNA was isolated using Isolate II RNA Micro Kit (Bioline, Heidelberg, Germany) according to the manufacturer's instructions. Equal RNA amounts were transcribed into cDNA by reverse transcriptase with the Precision nanoScript Reverse Transcription Kit (Primerdesign, Southhampton, UK) and carried out with a standard RT-PCR program (65°C, 5 min, 55°C, 20 min, 75°C, 15 min). For mRNA analysis in a quantitative real-time PCR, 5′FAM labeled testing probes of housekeeping genes GAPDH and RPL13A (Primer Design, Southampton, UK), TBX21 (Hs00203436_m1), BLIMP-1 (Hs00153357_m1), GATA3 (Hs00231122_m1), FOXP3 (Hs00203958_m1), SST2 (Hs01073297_m1), and ST2L (Hs00249389_m1) were used in the complete reaction mixtures. All probes were purchased from Applied Biosystems (Foster City, CA, USA) in a final concentration of 250 nM. The qRT-PCR were performed in technical duplicates with 5 μl Precision FAST 2x- qPCR Master-Mix (Primer Design, Southampton, UK), 3.5 μl H_2_O, 0.5 μl 5′FAM marked testing probe and 1 μl of cDNA. The following program was used: 95°C for 5 min, then alternating 3 s with 95°C and 30 s of 60°C (40x). Relative mRNA expression was calculated based on the normalized ratio of the non-regulated expression of GAPDH and RPL13A and the 2^−ΔCt^ method.

### Ethical Approvement

Serum samples and PBMC were isolated from buffy coats obtained drawn from anonymous healthy blood donors of the blood donation center DRK-Blutspendedienst Baden-Württemberg-Hessen, Institut für Transfusionsmedizin und Immunhämatologie Frankfurt am Main, Frankfurt, Germany. Prior blood sampling, all participants routinely gave written informed consent. According to the institutional ethics committee of the Goethe University Hospital, Frankfurt, Germany, and the local legislation, the need for an additional consent concerning the here presented experiments is not required, as the buffy coats were used anonymously for *in vitro* assays with no link to patient data.

### Statistical Analysis

Statistical analysis and data presentation were performed using GraphPad Prism 6 software (La Jolla, CA, USA). Appropriate statistical tests for multiple comparisons (RM one-way ANOVA and Friedman test for matched data, one-way ANOVA, Kruskal–Wallis for data not matched) or Wilcoxon matched-pairs signed rank test for matched *t*-test were used upon statistical testing of normal distribution with ns for *p* > 0.05, ^*^/# for *p* ≤ 0.05, ^**^/^##^ for *p* < 0.01, ^***^/^###^ for *p* < 0.001, and ^****^/^####^ for *p* < 0.0001. Asterisks (^*^) show comparisons as indicated, hashtags (#) to untreated controls. All data are represented as mean ± SD.

## Results

### Limited Bioactivity of IL-33 in Blood

**Figure 1:**
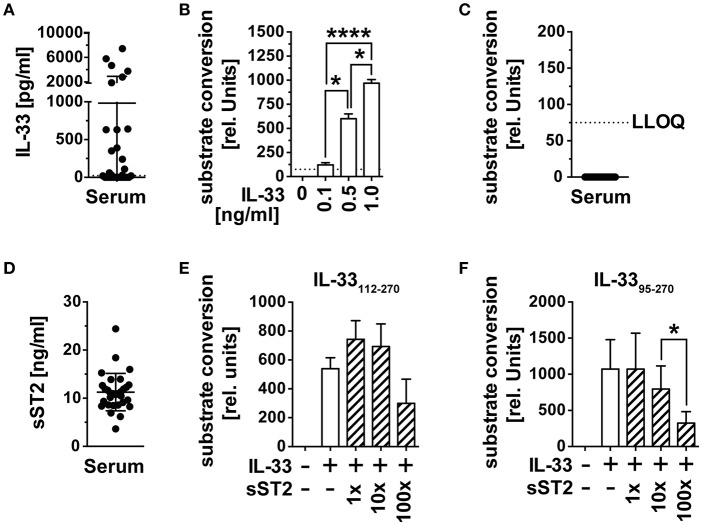
IL-33 detected in serum is biologically inactive. **(A)** IL-33 concentrations were determined in serum samples of *n* = 30 healthy male blood donors by ELISA. **(B)** The HEK293-ST2L reporter system was validated by stimulation with known concentrations of recombinant IL-33 before **(C)** measurement of bioactive IL-33 in serum. **(D)** Soluble ST2 (sST2) concentrations were determined in the same serum samples by ELISA. Blocking capacity of 1x, 10x, and 100x excess of sST2 toward recombinant **(E)** IL-33_112−270_ and **(F)** IL-33_95−270_ was assessed within the HEK293-ST2L bioactivity assay. Bioactivity of IL-33 within the HEK293-ST2L reporter system was indirectly determined by a dose-dependent increase of a converted substrate by a secreted alkaline phosphatase (SEAP). Data are shown as mean ± SD of *n* = 30 serum samples or *n* = 3–4 independently performed experiments with recombinant cytokines. *p ≤ 0.05, ****p < 0.0001 using *RM* one-way ANOVA with Tukey's post-test. LLOQ, lowest level of quantification; rel, relative.

The alarmin IL-33 has recently gained importance as an indicator of tissue damage and prognostic marker for the prognosis of cancer diseases. Its systemic role in the activation and recruitment of cytotoxic T cells to tumor tissue remains unclear, as bioactivity of IL-33 detected in blood by ELISA is missing. We therefore checked whether circulating immunoreactive IL-33 is biologically active in serum and first assessed IL-33 concentrations of serum samples from *n* = 30 healthy male blood donors (mean age 56.4 years, range 28–72) by ELISA. Immunoreactive IL-33 protein concentrations varied among these control samples, including 12 samples with no detectable (lowest level of quantification LLOQ 23 pg/ml), 12 with IL-33 concentrations between 23 and 64 pg/ml and six with IL-33 concentrations between 1,870 and 7,440 pg/ml (Figure 1A). We validated the functionality of the reporter system HEK293-ST2L for bioactive IL-33 with known concentrations of the recombinant cytokine (Figure 1B). Bioactivity was detected by indirect measurement of IL-33 induced NF-κB activity, which subsequently initiates expression of secreted alkaline phosphatase (SEAP) and conversion of an exogenously added substrate. In all cases, the corresponding IL-33 bioactivity was below the limit of detection of 75 relative units, corresponding to 75 pg/ml bioactive IL-33 (Figure 1C). Detection of soluble decoy receptor ST2 (sST2) by ELISA revealed high concentrations of 11.27 ± 3.89 ng/ml (Figure 1D). In order to evaluate if sST2 was entirely responsible for inhibition of IL-33 bioactivity in blood, we co-incubated 0.5 ng/ml of recombinant bioactive IL-33_112−270_ (Figure 1E) and IL-33_95−270_ (Figure 1F) with 1x, 10x, or 100x molar excess of sST2. Interestingly, sST2 failed to inhibit bioactivity of IL-33_112−270_. Bioactivity of IL-33_95−270_ was only significantly dampened upon co-incubation with 100x excess (mean 324.3 ± 158 rel. units) compared to 10x excess of sST2 (mean 795.2 ± 320.3 rel. units). The constitutively high concentrations of sST2 in blood implicate an important role of sST2 as a barrier of IL-33 bioactivity in blood. On the other hand, these experiments also showed a reduced blocking capacity of sST2 in competition to ST2L within the bioactivity reporter assay. Additionally, exclusively unbound, free IL-33 is detectable by commercially available ELISA as binding by sST2 leads to an inaccessibility of the epitopes necessary for antibody binding. We thus suggested that although sST2 represents a major regulator of IL-33 bioactivity in blood, IL-33 bioactivity might primarily be inhibited by intra-molecular mechanisms promptly upon release from cellular sources.

### Human Plasma Dampens Bioactivity of Recombinant IL-33

**Figure 2:**
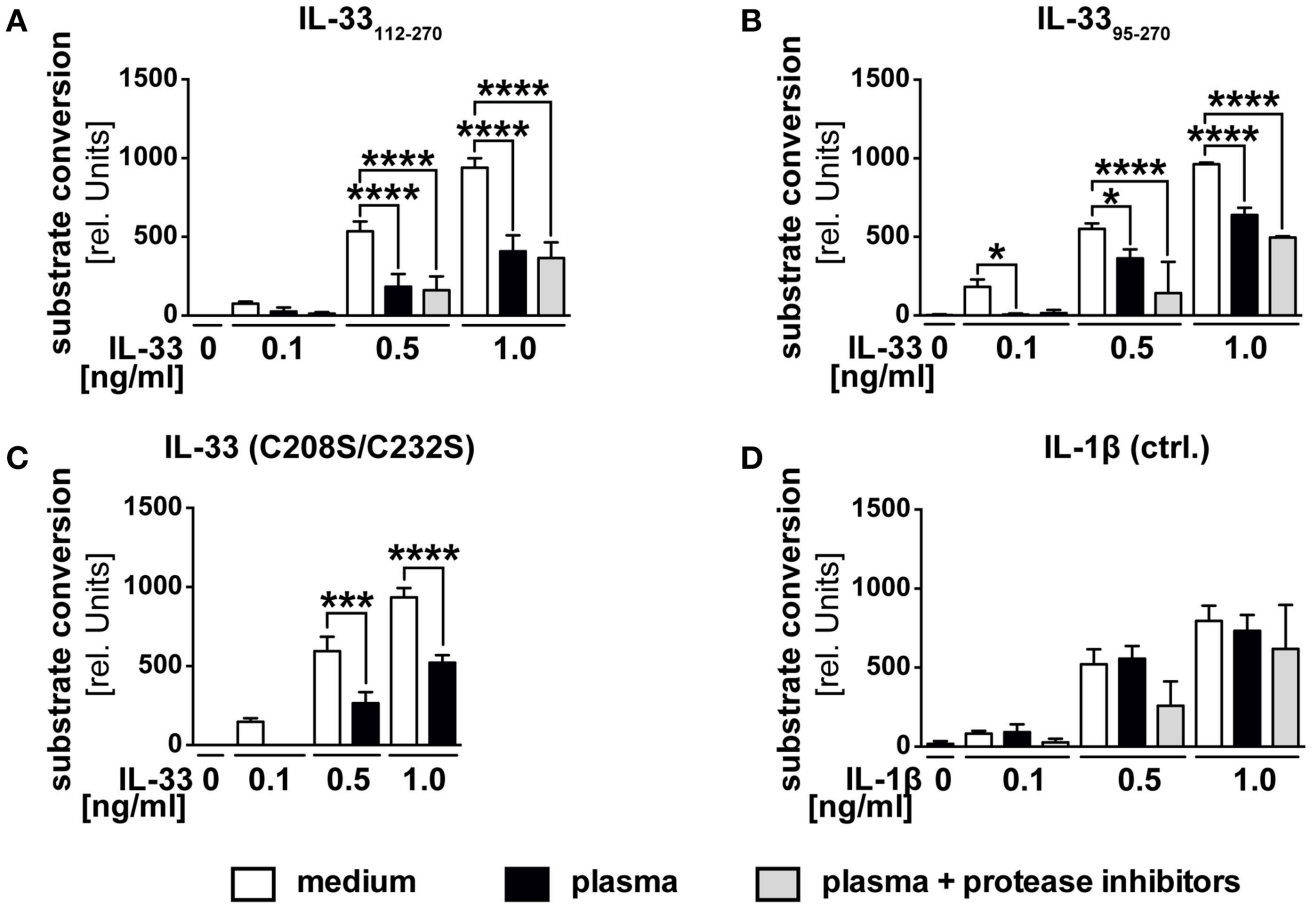
Human plasma negatively regulates IL-33 bioactivity. HEK293-ST2L reporter cells were cultured with medium as control or human plasma obtained from healthy blood donors. HEK293-ST2L cells were stimulated with 0.1, 0.5, or 1.0 ng/ml of IL-33 isoforms **(A)** IL-33_112−270_, **(B)** IL-33_95−270_, **(C)** oxidation resistant mutant C208S/C232S, or **(D)** IL-1β as control. Human plasma was added either natively or protease-inhibited. Viability of cells was examined by MTT viability assay, revealing no cytotoxic effects of plasma samples (data not shown). **(A–D)** Data are shown as mean ± SD of *n* = 3–10 different plasma samples with at least *n* = 3 independently performed experiments. *p ≤ 0.05, ***p < 0.001, and ****p < 0.0001 using one-way ANOVA with Tukey's post-test.

We postulated that the function of decoy receptor sST2 only represents a secondary mechanism to prevent a function of IL-33 in blood, but assumed that especially in the presence of ST2L, primarily intra-molecular mechanisms limit the range and duration of IL-33 bioactivity. We therefore investigated whether the observed downregulation in blood was caused by proteolytic processing or oxidation. We generated recombinant IL-33_95−270_, which represents IL-33 *in vivo* generated by mast cell-derived proteases and verified its bioactivity. Stimulation of HEK293-ST2L with IL-33_95−270_ led to a time-dependent increase of substrate conversion by SEAP (Supplementary Figure 1). While the presence of extracellular full-length IL-33 is unlikely, we aimed at artificially mimicking the *in vivo* situation by analysis of different size-reduced, mature IL-33 isoforms. According to our previous data (Figure 1), we expected an inactivation of recombinant bioactive IL-33 in serum. To challenge this hypothesis, we treated HEK293-ST2L cells with native human plasma and recombinant bioactive IL-33_112−270_ (Figure 2A), IL-33_95−270_ (Figure 2B), oxidation resistant mutant C208S/C232S (Figure 2C) as well as exogenous IL-1β as control for the experimental setting (Figure 2D). We used plasma to avoid a negative impact of coagulation on the cell-based reporter system. Bioactivities of IL-33_112−270_, IL-33_95−270_, but not IL-1β, were significantly downregulated upon incubation with native plasma in comparison to medium controls. By addition of protease inhibitors, we assured inhibition of inactivating proteolytic cleavage by blockade of serine, cysteine, aspartic, and aminopeptidases. Addition of protease inhibitors failed to rescue IL-33 bioactivity, hence excluding proteases as the effectors of bioactivity downregulation in plasma. Then we exogenously added an IL-33 mutant reported to be resistant to oxidation (Figure 2D). Bioactivity of IL-33 C208S/C232S was significantly reduced at a level comparable to controls not protected against oxidation, excluding oxidation as a cause of bioactivity downregulation as well (Figure 2C). The precise mechanism leading to the observed inhibition of exogenous, bioactive IL-33 in plasma needs further investigation. Nevertheless, these data implicate highly complex properties of plasma. We suggest that a combination of mechanisms on the molecular intrinsic level, as well as by sST2 lead to a limited bioactivity of IL-33 in blood. These observations strongly support previous suggestions of a local function of IL-33 released from necrotic cells in tissue and in the close environment of responsive cells.

### Nutrient Deprivation Mediates Expression of ST2L and Downregulation of CD8

**Figure 3:**
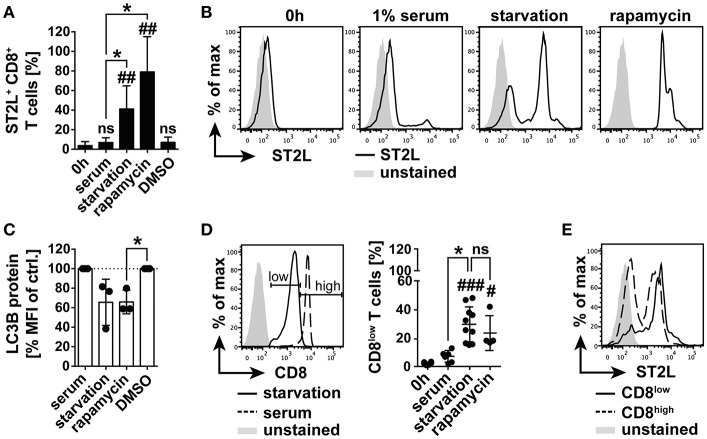
Nutrient deprivation mediates emergence of ST2L^+^ CD8^low^ T cells. **(A)** Expression of ST2L was determined on CD8^+^ T cells after isolation, upon 20 h cultivation with 1% human serum or under serum deprived conditions (starvation). Inhibition of mTOR was achieved by treatment with rapamycin in serum with DMSO in serum as control (DMSO). **(B)** Exemplary histograms of CD8^+^ T cells after isolation (0 h) or cultured with different conditions for 20 h. **(C)** Turnover of intracellular autophagy marker LC3B was assessed by flow cytometry in the respective treated CD8^+^ T cells. **(D)** Exemplary histograms showing the gating strategy for discrimination of CD8^high^ T cells and CD8^low^ T cells emerging upon serum deprivation. Percentage of CD8^low^ of total CD8^+^ T cells was assessed by flow cytometry. **(E)** Representative Data for ST2L expression on CD8^low^ and CD8^high^. Data are shown as mean ± SD of **(A,D)** 0 h *n* = 6, starvation *n* = 10, serum *n* = 7, rapamycin *n* = 4, DMSO *n* = 4 or **(C)** *n* = 3 per treatment from different donors and at least *n* = 3 independently performed experiments. *p ≤ 0.05, ##*p* < 0.01,###*p* < 0.001, and *p* < 0.0001 using Kruskal–Wallis test with Dunn's post-test for **(A,D)** or one-sample *t-*test for **(C)**. #for comparisons to the 0 h time point, *for comparisons as indicated.

Within our study, we had gained insight into the regulation of IL-33 bioactivity in blood. We were however further interested in the regulation of IL-33 responsivity of CD8^+^ T lymphocytes, as those represent a central cellular component in T cell mediated cytotoxicity and tumor immunity. IL-33 has been reported to be highly expressed in tumor tissue, in which nutrient gradients cause necrotic areas and release of bioactive IL-33. The role of IL-33 in tumor immunity is still highly controversial. Thus, in a controlled system we investigated the impact of bioactive IL-33 on the activation and polarization of CD8^+^ T cells. In order to mimic the harsh conditions of the tumor microenvironment, we additionally investigated the effects of nutrient deprivation. We analyzed expression of ST2L on selectively isolated CD8^+^ T cells and cultivated the cells with or without human serum. We found that starvation induced a significant percentage of ST2L^+^ CD8^+^ T cells (41.2 ± 23.7%) within the total CD8^+^ T cell population compared to the percentage of ST2L^+^ CD8^+^ T cells directly after isolation (3.7 ± 4.1%) or cultured in serum (5.5 ± 3.5%). Although a complexity of mechanisms is affected during nutrient deprivation, we pharmacologically induced conditions resembling nutrient deprivation by inhibition of mechanistic target of Rapamycin (mTOR) using rapamycin. Addition of rapamycin to CD8^+^ T cells cultured in serum induced a significant expression of ST2L comparable to the effects observed during starvation (79.1 ± 36.0%) (Figures 3A,B). As described, nutrient deprivation leads to inhibition of mTOR, and subsequent initiation of autophagy. Microtubule-associated protein light chain 3B (LC3B) degradation is an indicator for autophagy. We assessed turnover of LC3B in CD8^+^ T cells upon starvation, treatment with rapamycin, and cultivation with serum by measurement of the expression levels of LC3B as mean fluorescence intensity (MFI). LC3B MFI was reduced upon starvation (65.6 ± 23.7% of control) and was significantly reduced upon treatment with rapamycin (65.9 ± 12.1% of control) (Figure 3C). Importantly, we observed that starvation led to dampening of CD8 expression, resulting in emergence of defined populations designated CD8^low^ and CD8^high^. The percentage of CD8^low^ T cells was significantly raised by starvation (30.0 ± 12.3%) and treatment with rapamycin (23.9 ± 12.1%) compared to cell cultured in serum (Figure 3D). Considering the total CD8^+^ T cell population, CD8^low^ T cells were identified as the main population expressing ST2L (Figure 3E). In this experiment, we demonstrate for the first time that ST2L expression is induced on CD8^+^ T cells during nutrient deprivation, which may be indicative for the microenvironment of solid tumors. Moreover, we describe CD8 downregulation mediated by starvation.

### ST2L^+^ CD8^low^ Are Transitional CCR7^+^ T Cells

**Figure 4:**
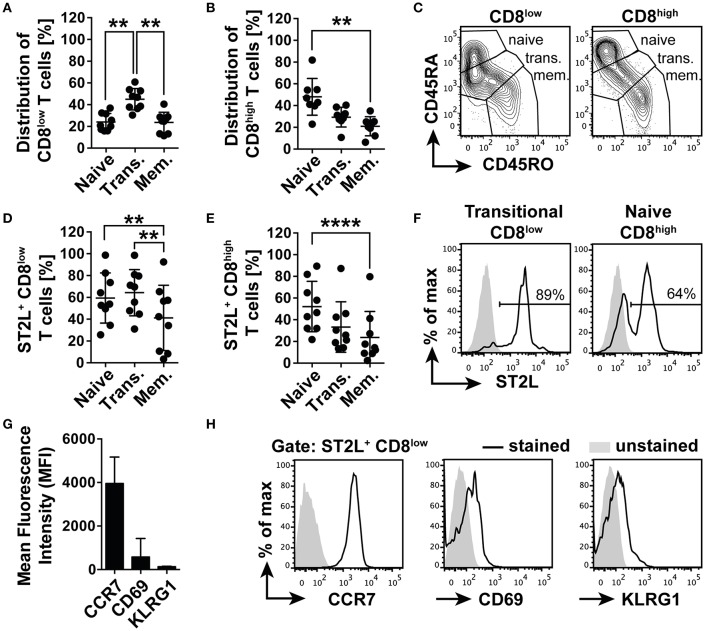
ST2L^+^ CD8^low^ T cells are transitional CCR7^+^ T cells. Distribution of naïve (CD45RA^+^RO^−^), transitional (Trans., CD45RA^+^RO^+^) and memory type (Mem., CD45RA^−^RO^+^) subpopulations of **(A)** CD8^low^ and **(B)** CD8^high^ T cells determined by flow cytometry. **(C)** Representative data for gating strategy of naïve, transitional, and memory type CD8^low^ and CD8^high^. Percentage of ST2L expressing naïve, transitional, or memory type **(D)** CD8^low^, and **(E)** CD8^high^. **(F)** Representative histograms of ST2L expression of transitional CD8^low^ and naïve CD8^high^. **(G)** Mean fluorescence intensity of ST2L^+^ CD8^low^ expressing CCR7, CD69, and KLRG1 and **(H)** representative flow cytometry data. Data are shown as mean ± SD of **(A–F)** *n* = 9 or **(G,H)** *n* = 5 of different donors with at least *n* = 3 independently performed experiments. **p < 0.01, ****p < 0.0001 using **(A,B)** Kruskal–Wallis test with Dunn's post-test or **(D,E)** Friedman test with Dunn's post-test.

Before functional characterization, we first described the phenotype of the emerging CD8 populations in order to understand the connection between nutrient deprivation and the possible function of ST2L expression on CD8^low^ T cells. For this purpose, we analyzed the cell surface expression and distribution of naïve T cell marker CD45RA and memory T cell marker CD45RO. Populations with the expression patterns CD45RA^+^RO^−^ were defined as naïve, CD45RA^+^RO^+^ co-expressing cells as transitional and CD45RA^−^RO^+^ as memory type CD8^+^ T cells. The analysis revealed that CD8^low^ T cells contained a significantly higher percentage of transitional cells (45.0 ± 9.8%), dominating the fractions of naïve (24.1 ± 7.9%) and memory CD8^low^ T cells (23.6 ± 9.5%) (Figures 4A,C). However, the CD8^high^ population was dominated by naïve CD45RA^+^RO^+^ T cells (48.0 ± 16.9%), significantly exceeding the percentage of memory type CD45RA^−^RO^+^ T cells (21.0 ± 8.8%) (Figures 4B,C). Corresponding to the distribution of naïve, transitional, and memory type T cells within the CD8^low^ and CD8^high^ subpopulations, we identified naïve (59.4 ± 23%), and especially transitional CD8^low^ (64.3 ± 21.2%) as the main populations expressing ST2L, compared to the population defined as memory type CD8^low^ T cells (41.2 ± 30%) (Figures 4D,F). A higher percentage of naïve CD8^high^ T cells was shown to express ST2L (52.2 ± 23.2%) compared to transitional (33.3 ± 23.3%) and memory type cells (23.6 ± 24%) (Figures 4E,F). We suspected that CD8^low^ T cells were in a transitional position awaiting antigen encounter and suspected a high migratory potential of these cells to secondary lymphoid organs. In accordance with this notion, we determined a high expression of chemokine homing receptor CCR7 and low expression of both activation marker CD69 and cytotoxicity marker KLRG1 on ST2L^+^ CD8^low^ (Figures 4G,H). Taken together, we identified ST2L^+^ CD8^low^ emerging upon nutrient deprivation as undifferentiated transitional CCR7^+^ CD69^low^ KLRG1^low^ type CD8^+^ T cells.

### IL-33 Suppresses TCR-Independent Pro-inflammatory Immunity by Induction of an Anti-inflammatory GATA-3 and FoxP3 Driven Differentiation Program

**Figure 5:**
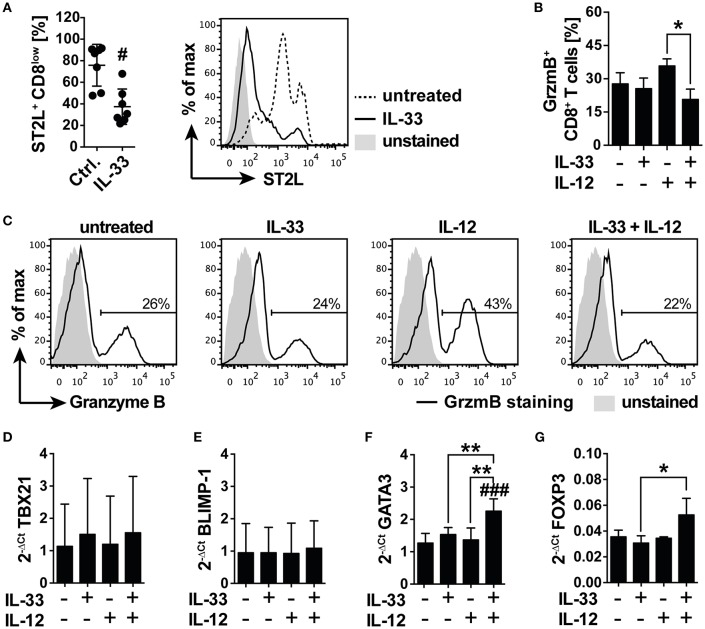
IL-33 inhibits IL-12 induced granzyme B expression in a nutrient deprived environment. **(A)** Serum deprived CD8 T cells were treated with 20 ng/ml IL-33 prior determination of abundance of ST2L^+^ CD8^low^ cells by flow cytometry. Representative flow cytometry histograms showing ST2L expression on CD8^low^ T cells treated with IL-33 compared to untreated control. Ctrl. = untreated starved CD8^+^ T cells. **(B)** Percentage of CD8 T cells expressing intracellular Granzyme B (GrzmB) upon treatment with IL-33 [20 ng/ml], IL-12 [5 ng/ml], or the combination of both. **(C)** Representative data showing the modulation of intracellular GrzmB expression by IL-33 and IL-12. qRT-PCR relative expression (2^−ΔCt^) of **(D)** *TBX21 (T-bet)*, **(E)** *BLIMP-1*, **(F)** *GATA3*, and **(G)** *FOXP3*. The expression levels were normalized to the housekeeping genes *GAPDH* and *RPL13A*. Data are shown as mean ± SD of **(A)** *n* = 7, **(B)** *n* = 4, **(D,E)** *n* = 4 and **(F,G)** *n* = 3 of different donors with at least *n* = 3 independently performed experiments. *,^#^*p* ≤ 0.05, **p < 0,01, and ^###^*p* < 0.001 using **(A)** Wilcoxon matched-pairs signed rank test, **(B)** Friedman test with Dunn's post-test, or **(D–G)** RM one-way ANOVA with Bonferroni's post-test. Hashtags (#) indicate significance compared to the untreated control, asterisks (*) indicate significance as indicated.

We next asked if IL-33 supports or counter-regulates TCR-independent inflammation in starved CD8^+^ T cells and expected that downregulation of CD8 led to a desensitization toward antigen-independent activation. We cultured isolated human CD8^+^ T cells under serum deprivation in order to induce the previously observed expression of ST2L. Cell surface analysis revealed a significant reduction in the percentage of ST2L^+^ CD8^low^ T cells upon treatment with 20 ng/ml IL-33 (37.5 ± 16.4%; Ctrl. 75.9 ± 19.4%), indicating responsiveness of the target cells to the cytokine (Figure 5A). To assess the cytotoxic potential of the treated CD8^+^ T cells, we monitored the intracellular expression of granzyme B (GrzmB) by flow cytometry. Treatment with IL-12, but not IL-33, induced an increase in intracellular GrzmB protein expression. Importantly, treatment with the combination of IL-12 and IL-33 significantly reverted the IL-12 induced upregulation of GrzmB expression (Figures 5B,C). mRNA analysis of starved CD8^+^ T cells treated with IL-33, IL-12 or in combination disclosed no differences in the mRNA expression of cytotoxic T cell lineage transcription factor *TBX21* (T-bet) (Figure 5D) or memory effector transcription factor *BLIMP-1* (Figure 5E). Notably, co-stimulation with IL-33, and IL-12 significantly induced mRNA expression of Th2 lineage marker *GATA3* (Figure 5F) and regulatory *FOXP3* (Figure 5G). The increased mRNA expression of GATA3 and FoxP3 was confirmed on protein levels by flow cytometry analysis, showing that intracellular GATA3, and FoxP3 protein expression was significantly induced in CD8^+^ T cells (Supplementary Figures 2A–D). Additionally, the ratio of CD8^high^ to CD8^low^ T cells was significantly reduced upon co-stimulation with IL-33 and IL-12. Speculating that CD8^high^ T cells represented effector cells, these data suggested a regulatory function exerted by CD8^low^ directed against the effector activity of CD8^high^ (Supplementary Figure 2E).

### IL-33 Signaling Overcomes Immunosuppression of Nutrient Deprivation by Co-stimulation of TCR-Mediated T-Bet Dependent Effector Functions

**Figure 6:**
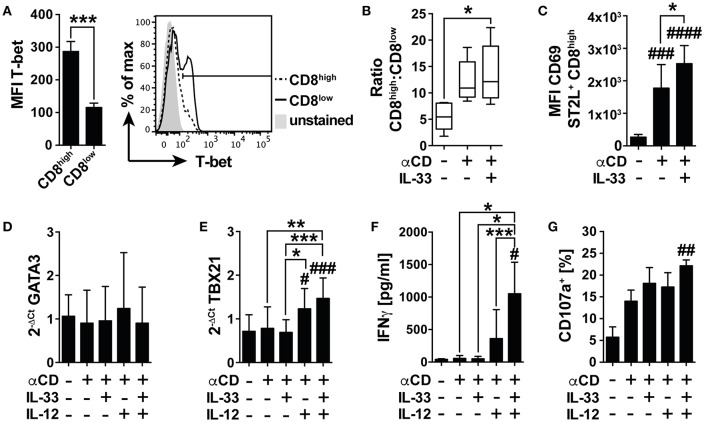
IL-33 supports TCR-dependent pro-inflammatory effector functions. **(A)** Mean fluorescence intensity (MFI) of intracellularly expressed T-bet in CD8^high^ and CD8^low^ T cells and representative histogram. **(B)** Ratio of CD8^high^ to CD8^low^ T cells or **(C)** MFI of CD69 expression on ST2L^+^ CD8^high^ during serum deprivation without treatment, with TCR-specific αCD2/CD3/CD28 stimulation and co-treatment of IL-33 and αCD2/CD3/CD28 for 20 h. qRT-PCR relative expression (2^−ΔCt^) of **(D)** *GATA3* and **(E)** *TBX21 (T-bet)*. The expression levels were normalized to the housekeeping genes *GAPDH* and *RPL13A*. **(F)** Concentrations of secreted IFNγ were determined in the supernatants of untreated and stimulated cells, respectively. **(G)** For determination of cytotoxicity, percentage of CD8^+^ T cells expressing CD107a on the cell surface was determined by flow cytometry. Data are shown as mean ± SD of *n* = 4–5 different donors with at least *n* = 3 independently performed experiments. *,^#^*p* ≤ 0.05, **,^##^*p* < 0.01, and ***,^###^*p* < 0.001 and ^####^*p* < 0.0001 using **(A)** student's paired *t-*test, **(B,G)** Friedman test with Dunn's posttest, **(C–F)** RM one-way ANOVA with Tukey's post-test. Hashtags (#) indicate significance compared to the untreated control, asterisks (*) show significance as indicated.

Under nutrient deprivation, IL-33 induced mRNA expression of *GATA3*, and *FOXP3* in CD8^low^ T cells, implicating differentiation into an anti-inflammatory, regulatory phenotype following TCR-independent stimulation. We were furthermore interested in the effects of IL-33 on the TCR-induced activity of CD8^+^ T cells and first assessed the intracellular expression of T-bet as a master regulator of pro-inflammatory immunity in CD8^high^ and CD8^low^ T cells (Figure 6A). The expression level of T-bet in CD8^high^ significantly exceeded the low expression of T-bet detected in CD8^low^, implicating that CD8^high^ T cells were effector T cells, and regarding expression of ST2L on this population, we asked whether IL-33 mediated pro-inflammatory effects in CD8^high^, respectively. We therefore next determined the ratio of CD8^high^ to CD8^low^ T cells. Treatment of αCD and IL-33 in combination significantly raised the ratio of CD8^high^ to CD8^low^ T cells (Figure 6B). Correspondingly, IL-33 significantly increased expression of activation and tissue residency marker CD69 on ST2L^+^ CD8^high^ upon TCR-dependent activation by αCD (Figure 6C). Evaluating the mRNA expression of *GATA3* (Figure 6D), and *TBX21* (T-bet mRNA) (Figure 6E), we found that while *GATA3* was not regulated, *TBX21* expression was significantly increased during co-treatment with IL-33, IL-12, and αCD in spite of serum deprivation. These results indicate that IL-33 does not inhibit TCR-induced inflammation during nutrient deprivation, but selectively inhibits TCR-independent activation. We further demonstrated that analogous to the murine model, IL-33 synergizes with the TCR and IL-12 signaling to promote IFNγ secretion by human CD8^+^ T cells (Figure 6F). IFNγ secretion went along with significantly increased degranulation of the CD8^+^ T cells upon co-activation with αCD, IL-33, and IL-12, which was determined by measurement of CD107a translocated to the cell surface during stimulation (Figure 6G, Supplementary Figure 3A). Corresponding to our data showing that CD8^high^ highly expressed T-bet, we also identified CD8^high^ to express higher levels of CD107a on the cell surface than CD8^low^ (Supplementary Figure 3B). These data show that TCR stimulation overcomes the immunosuppressive effects of nutrient deprivation dependent on co-stimulation with IL-33 and IL-12. We provide evidence for a role of IL-33 in the regulation of CD8 activity during nutrient deprivation, in which IL-33 modulates the cytotoxic CD8 T cell activity by regulation of lineage-specific differentiation programs.

### TCR Dependent Activation Initiates Differential mRNA Expression of IL-33 Decoy Receptor sST2 in PBMC

**Figure 7:**
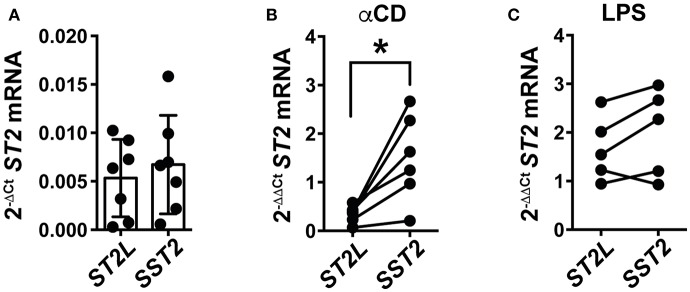
TCR-dependent stimulation of PBMC induces expression of *SST2* mRNA. qRT-PCR relative expression (2^−ΔCt^) of **(A)** *ST2L* and *SST2 mRNA* in untreated PBMC and upon **(B)** TCR-dependent activation by αCD2/3/28 or **(C)** treatment with LPS. The expression levels were normalized to the housekeeping genes *GAPDH* and *RPL13A*. Data are shown as mean ± SD of **(A)** *n* = 7, **(B)** *n* = 5, and **(C)** *n* = 6 independently performed experiments. *p ≤ 0.05 using Wilcoxon matched-pairs rank test.

Our study sought to explain the differential regulation of IL-33 signaling by molecular mechanisms and on the cellular level, at once representing the role of IL-33 in blood and in tissue, respectively. As IL-33 potently supports TCR-dependent activation of CD8^+^ lymphocytes, we hypothesized that activation of T cells would subsequently lead to induction of counter-regulatory mechanisms to prohibit uncontrolled, IL-33 mediated inflammation. We herein focused on the differential expression of *ST2L* and *SST2* mRNA in peripheral blood mononuclear cells (PBMC) upon pro-inflammatory stimulation. For this, we again chose αCD treatment for specific activation of T lymphocytes, and lipopolysaccharide (LPS) for stimulation of cellular components of the innate immune system as a control. Untreated PBMC constitutively expressed low levels of *ST2L* and *SST2* mRNA (Figure 7A). T cell receptor (TCR)-dependent stimulation with αCD induced a strong and significantly higher expression of *SST2* mRNA compared to *ST2L* mRNA (Figure 7B), while LPS resulted in an increased and equal expression of both *ST2L* and *SST2* mRNA (Figure 7C). In line with our previous results, we suggest that IL-33 supports the TCR-dependent activity of T lymphocytes, but concurrently, limitation of time and range of IL-33 effects in blood is ensured by expression of sST2.

## Discussion

IL-33 has been widely proven to be a driver of innate immunity, inflammation, and atopic diseases (23, 24). Although the role of IL-33 in tumor immunity is discussed controversially, IL-33 detected in serum has been proposed as a prognostic biomarker for different cancer diseases (25–27). In this study, we therefore, first questioned the systemic function of the alarmin, then determined conditions favoring IL-33 signaling through expression of ST2L, and identified the relevance of IL-33 in the differentiation of CD8^+^ T lymphocytes under *in vitro* conditions artificially mimicking a tumor microenvironment.

### IL-33 in Blood Is Biologically Inactive

Bioactivity of IL-33 as an alarmin is highly regulated by modulation of ST2L expression on the cellular level, by intra-molecular mechanisms increasing or inhibiting bioactivity (e.g., proteolytic processing) and by constitutive expression of decoy receptor sST2 in blood. We suggested that compartment specific activation and inactivation limit IL-33 bioactivity to the close environment of its cellular sources before to avoid systemic effects. In a first step, we investigated whether IL-33 detected in the serum of healthy donors is biologically active and thus contributes to a systemic activation or recruitment of CD8^+^ T cells. IL-33 modulates the tumor-associated inflammatory microenvironment by activation of the NF-κB pathway and Th1 transcription factor T-bet, promoting proliferation, activation and infiltration of CD8^+^ T cells and NK cells (15). We therefore made use of and evaluated the functionality of an IL-33 sensitive NF-κB reporter gene detection system to investigate the capacity of IL-33 detected in serum to promote inflammation (Figure 1). Additionally, we generated a bioactive isoform IL-33_179−270_, and inactive IL-33_95−270_ to investigate whether those isoforms are differentially modulated. IL-33 detected in serum samples of healthy donors by ELISA lacked bioactivity, as they failed at activating the NF- κB pathway (Figures 1A,C). Bioactivity of IL-33 is mainly determined by the accessibility of the binding sites located within the IL-1-like cytokine domain and is strongly regulated by a variety of mechanisms, including sST2. We detected high concentrations of sST2 in the same serum samples (Figure 1D) but observed that bioactivity of IL-33_95−270_ was only downregulated upon co-incubation with 100x excess of soluble receptor. In contrast, Lingel et al. determined that sST2 binds to IL-33_112−270_ with high affinity and a dissociation constant of 4 nM (28). Although further studies are inevitable, we suspected that the blocking capacity of sST2 was reduced due to a competition to ST2L and co-receptor IL-1RaCP. Furthermore, we found that IL-33 complexed by sST2 is not detectable by ELISA presumably due to a limited accessibility of the antibody-specific epitopes (data not shown). Assuming an exclusive function of sST2 as a decoy receptor, the observed high expression in blood would represent a mechanism avoiding systemic effects of IL-33. As sST2 exhibited a low binding capacity of IL-33 in the presence of ST2L, we suspected that primarily proteolytic processing or oxidation might limit IL-33 bioactivity directly upon release. Interestingly, bioactivity of recombinant exogenous IL-33 was massively downregulated in plasma (Figure 2). “Mature” IL-33 isoforms exert strong bioactive features and are generated by pro-inflammatory proteases upon cleavage within the N-terminal nuclear localization or central domain (amino acid residues (aa 1–111) (10, 29). Although self-generated IL-33_95−270_ exhibited a higher bioactivity than IL-33_112−270_ within the reporter assay we performed, bioactivities of both isoforms were equally downregulated in human plasma. Long-term exposure of IL-33 to serine protease PR3 located in blood was described to inactivate IL-33 by cleavage within the IL-1-like cytokine and receptor binding domain (aa 112–270) (30). Inhibition of serine, cysteine, aspartic and aminopeptidases however failed at reverting the effect, excluding proteases as inactivators (Figures 2A,B). Cohen et al. described that IL-33 bioactivity is abrogated by oxidation (11). Still, the bioactivity of IL-33 resistant to oxidation was dampened in plasma (Figure 2C). The effect was exclusive for IL-33 and did not result from technical difficulties, as bioactivity of recombinant IL-1β used as a control was not affected in plasma (Figure 2D). Although the mechanism lying beyond the inactivation in blood remains to be elucidated, our results strongly implicate that IL-33 detected in serum represents an echo of inflammation, rather than a systemic activator. We propose that inactivation of IL-33 results from a combination of different mechanisms on different levels. As our data indicated that the blocking capacity of sST2 in the presence of ST2L was reduced, we suggest that primarily intra-molecular mechanisms, which yet remain to be elucidated, rapidly downregulate bioactivity of IL-33 in the presence of responsive cells. sST2 might hence represent a secondary barrier in blood.

### IL-33 Inhibits TCR-Independent CD8 Effector Activity During Nutrient Deprivation by Induction of a GATA3/FOXP3 Differentiation Program

Expression of ST2L on CD8^+^ T cells represents a mechanism regulating IL-33 bioactivity on a cellular level also in the context of tumor immunity, although the function of IL-33 in tumor immunity is currently controversially discussed. Based on our previous findings indicating inactivity of IL-33 in blood, we concluded that the role of IL-33 in adaptive tumor immunity would be limited to infiltrated or tissue resident effector CD8^+^ T lymphocytes. Activity and fitness of CD8^+^ effector T cells located in tumor tissue is highly influenced by impeded perfusion, oxygen, nutrient, and growth factor delivery (17, 31). Although generally excluded from peripheral tissues, naïve CD8^+^ T cells infiltrate CCR7-dependently and are activated in tumor mass (32, 33), which represents—in spite of the numerous immunosuppressive mechanisms—an attractive site of T cell priming (32, 34). Here, we first analyzed the role of IL-33 in the modulation of the TCR-independent inflammation in naïve CD8^+^ T cells. We purposely omitted the already widely discussed effects of tumor-associated cells promoting tumor growth to focus on the aspect of nutrient deprivation within tumor tissue and its impact on CD8 activity. Nutrient deprivation of CD8^+^ T cells massively induces cellular stress, apoptosis and an altered metabolism through influence of cytoplasmic nutrient sensors like mTOR (35). In this study, we observed a significant induction of ST2L and downregulation of CD8 expression in starved CD8^+^ T lymphocytes, naming the emerging population ST2L^+^CD8^low^ due to its distinctive phenotype (Figures 3A,D,E). Although not investigated in this study, it is tempting to speculate that ST2L is expressed via internal ribosome entry sites (IRES) under translation-inhibitory conditions such as starvation or hypoxia. This assumption is based on the finding of Kunze et al. that sST2 is expressed IRES-dependently during nutrient deprivation (36). To pharmacologically induce effects comparable to nutrient deprivation, we treated CD8^+^ T cells cultured in serum with rapamycin and showed that inhibition of mTOR resulted in similar phenotypic changes including ST2L expression and downregulation of CD8 (Figures 3A,D). Inhibition of mTOR followed by autophagy was described as beneficial for the generation of memory CD8 T cells and survival of effector cells (20, 37). We thus assessed degradation of LC3B as an indication for autophagy (38) and confirmed that autophagy was induced (Figure 3C). While the role of mTOR in T cell immunity is not yet clearly defined, evidence implicates that mTOR positively regulates effector and regulatory T cell lineage commitment (39, 40). Phenotypical characterization revealed that CD8^high^ T cells resembling lymphocytes cultured in serum mainly exhibited a naïve CD45RA^+^RO^−^ phenotype (Figures 4B,C). CD8^low^ T cells were characterized by low expression of both CD45RA and memory T cell marker CD45RO, which is defined as a transitional phase (41–43). Strikingly, expression of ST2L was highest on transitional CD8^low^ (Figures 4A,D). As nutrient deprivation leads to a reduced ability of lymphocytes to respond to pro-inflammatory stimuli and cytokines (44), we asked if CD8^low^ T cells were desensitized toward TCR-independent stimuli. ST2L^+^ CD8^low^ T cells disclosed high expression of homing receptor CCR7, indicating a migratory behavior to secondary lymphoid organs and lacked expression of markers associated to tissue residency or effector function (Figures 4G,H). As CD8^low^ T cells located in peripheral blood or tumor tissue have on the one hand been described as highly cytotoxic effector cells and on the other hand as T cells with decreased sensitivity and poor cytolytic potential, we questioned the function of CD8^low^ evolved from nutrient deprivation (45–49). IL-33 is highly expressed in diverse cancer tissues (50). The heterogeneity of tumor blood flow and growing tumor mass cause necrotic areas within tumor tissue or tumoral HEV, which represent sources of IL-33. Treatment of ST2L^+^ CD8^low^ T cells with IL-33 implicated responsiveness toward the ligand due to downregulation of ST2L indicating receptor internalization (Figure 5A). We observed that IL-33 reverted an IL-12 induced increase in intracellular GrzmB protein during nutrient deprivation (Figures 5B,C). IL-33 contributes to the differentiation of T cell subsets by regulation of the Th1, Th2, and Treg lineage-specifying transcription factors T-bet, GATA-3, and Foxp3 in a positive feedback loop (4). Our experiments disclosed that co-treatment of IL-33 and IL-12 in the absence of TCR activation led to increased expression of *GATA3* and *FOXP3* mRNA, suggesting induction of an anti-inflammatory, regulatory phenotype (Figures 5F,G). We additionally confirmed that the increase in *GATA3* and *FOXP3* mRNA corresponded to an increase in the protein expression of both transcription factors upon IL-33 and IL-12 stimulation. Furthermore, induction of the anti-inflammatory phenotype was accompanied by a reduction in the ratio of CD8^high^ to CD8^low^ T cells, suggesting a decreased number of effector T cells and predominance of CD8^low^ T cells (Supplementary Figure 2). Interestingly, GATA3 as a repressor of pro-inflammatory, T-bet dependent effector differentiation is involved in the dysfunction of tumor infiltrating CD8^+^ T cells (51, 52). T-bet mRNA *TBX21* and effector memory transcription factor *BLIMP-1* mRNA were correspondingly not regulated (Figures 5D,E). In accordance with our data, Guo et al. demonstrated that GATA3 and STAT5 drive a constitutively high expression of ST2L on Th2 cells and Tregs (53). We suggest that nutrient deprivation induces expression of ST2L. IL-33 signaling in turn initiates a GATA3/Foxp3 dependent differentiation program, which counteracts a TCR-independent stimulation of energy-consuming effector functions.

### IL-33 Supports TCR-Mediated Effector Functions in a T-Bet Dependent Manner

We and others have previously demonstrated that IL-33 synergizes with the TCR and IL-12 to synergistically induce IFNγ expression in murine CD8^+^ T cells (13, 14). We demonstrated that CD8^high^ T cells expressed significantly higher levels of transcription factor T-bet, a driver of inflammation (Figure 6A). We thus suspected ST2L^+^ CD8^high^ to constitute a population of effector CD8^+^ T cells, whereas ST2L^+^ CD8^low^ T cells exhibited anti-inflammatory functions during TCR-independent stimulation. In fact, in contrast to TCR-independent activation, TCR-specific stimulation (αCD2/3/28; αCD) in combination with IL-33 resulted in a significant increase in the ratio of CD8^high^ to CD8^low^ T cells (Figure 6B) and expression of activation and tissue residency marker CD69 on ST2L^+^ CD8^high^ T cells (Figure 6C). ST2L expression in CD4^+^ Th1 effector cells was proven to be dependent on T-bet and STAT4. ST2L-deficient effector cells in turn exhibited reduced IFNγ expression (54). We were therefore not surprised to find that—in accordance with our murine model—treatment with αCD, IL-33, and IL-12 induced mRNA expression of T-bet as a master regulator of pro-inflammatory effector functions and IFNγ secretion in the human model (Figures 6E,F) while in contrast to CD8^+^ T cells treated TCR-independently, *GATA3* mRNA was not regulated (Figure 6D). CD107a (Lamp-1) is a lysosomal glycoprotein located in the membrane of cytotoxic granules and is translocated to the cell surface as a result of degranulation, thus serving as a sensitive method for identification of cytotoxic T cells (55). Co-stimulation of the TCR with IL-33 and IL-12 led to a significant increase in CD107a detected on the cell surface of the isolated CD8^+^ T cells (Figure 6G, Supplementary Figure 3A). Corresponding to the high expression levels of T-bet in CD8^high^ T cells, we also found that CD8^high^ T cells were the major population expressing CD107a on the cell surface (Supplementary Figure 3B). Recognition and rejection of tumor cells by effector T cells is functionally impaired by signals from the tumor microenvironment (56). We emphasize that IL-33 on the one hand mediates anti-inflammatory immunity during nutrient deprivation, which is in line with the widely discussed function of IL-33 as a guardian of tissue homeostasis (57). On the other hand, IL-33 overcomes immunosuppression mediated by starvation and fuels potent pro-inflammatory cytotoxicity of CD8^+^ T cells by synergizing with IL-12 and the TCR.

### TCR-Dependent Stimulation Initiates Expression of Decoy Receptor *SST2* mRNA in PBMC

We further questioned whether TCR-dependent stimulation of T lymphocytes initiates a control mechanism regulating IL-33 bioactivity. mRNA of both *ST2L* and *SST2* were expressed in untreated PBMC at low levels (Figure 7A). We activated T cells by TCR-dependent stimulation with αCD (Figure 7B) and components of the innate immune system with LPS (Figure 7C). αCD treatment, but not LPS, induced a differential expression of *SST2* mRNA, which significantly exceeded expression of *ST2L* mRNA. Concurrently, previous studies demonstrated that sST2 expression is attenuated upon inhibition of NF-κB (58). sST2 has primarily been described as a decoy receptor to inhibit IL-33 bioactivity. Although to date not reported for IL-33 and sST2, binding of cytokines to their soluble receptors has however been reported to prolong the half-life of the respective circulating cytokines, and to increase bioactivity (59). We and others (60) suggest considering this possibility for IL-33 and sST2, which upon verification would require re-evaluation of conclusions drawn from high sST2 concentrations in blood and the systemic function of IL-33. Based on the acceptance of sST2 as a decoy receptor, Zhang et al. suggested that IL-33 sequestration by sST2 not only inhibits systemic effects of IL-33, but also promotes a pro-inflammatory Th1/Th17 response by affecting the balance to anti-inflammatory Th2/Treg cells (61).

The overall data presented in this study indicate a highly complex crosstalk of mechanisms regulating IL-33 bioactivity in blood and in tissue. While IL-33 seems to be inactive in blood, nutrient availability in tissue, the presence of pro-inflammatory cytokines, and TCR-signaling appear to be decisive for the function of IL-33 as a co-factor for CD8^+^ T lymphocytes. IL-33 either suppresses or enhances effector functions by initiating differentiation programs associated to cytotoxic or regulatory T cell lineages. We identified for the first time, to our knowledge, that nutrient deprivation induces responsiveness of CD8^+^ T cells to IL-33. Further research will focus on the connection between mTOR inhibition during starvation and expression of ST2L. As the here presented data implicate an interesting role of mTOR in the modulation of T cell dependent cytotoxicity and represents a potential pharmacological target, our *in vitro* data are to be validated in a patient study. Differences between healthy subjects and tumor patients might not only occur regarding the systemic activity and function of IL-33, but also with respect to the IL-33-dependent differentiation of pro- and anti-inflammatory CD8^high^ and CD8^low^ T cells. The distribution of ST2L^+^ effector CD8^high^ and transitional or memory CD8^low^ T cells in tumor tissue might therefore reveal essential findings contributing to the understanding of the IL-33/ST2L axis in cancer immunity.

## Data Availability

All datasets generated for this study are included in the manuscript and/or the Supplementary Files.

## Author Contributions

CD performed the experiments, wrote the manuscript, performed statistics, and designed the figures. FO planned and performed the ELISA experiment for IL-33 and sST2 detection in samples of healthy volunteers. KS and MH helped in planning the experiments, and by writing the manuscript. AE and MP generated the recombinant IL-33 isoforms aa95-270 and aa179-270. JP provided the basic laboratory equipment. HR had the idea, designed, and supervised all experiments, checked all data in detail and finalized the manuscript.

### Conflict of Interest Statement

The authors declare that the research was conducted in the absence of any commercial or financial relationships that could be construed as a potential conflict of interest.

## Abbreviations

BLIMP-1: PR domain zing finger protein 1
GATA3: GATA binding protein 3
NF-kB: nuclear factor “kappa-light-chain-enhancer” of activated B cells
PI-3K: Phosphoinositide 3-kinase
T-bet, TBX21: T-box transcription factor 21
Th: T helper cell.
