# Supplementary material for: Tissue Cytokine IL-33 Modulates the Cytotoxic CD8 T Lymphocyte Activity During Nutrient Deprivation by Regulation of Lineage-Specific Differentiation Programs
Source: Front Immunol. 2019 Jul 24;10:1698. doi: 10.3389/fimmu.2019.01698 (PMC6667839; doi:10.3389/fimmu.2019.01698)
Supplement: Supplementary file 1 [file Data_Sheet_1.docx]

Supplementary Material


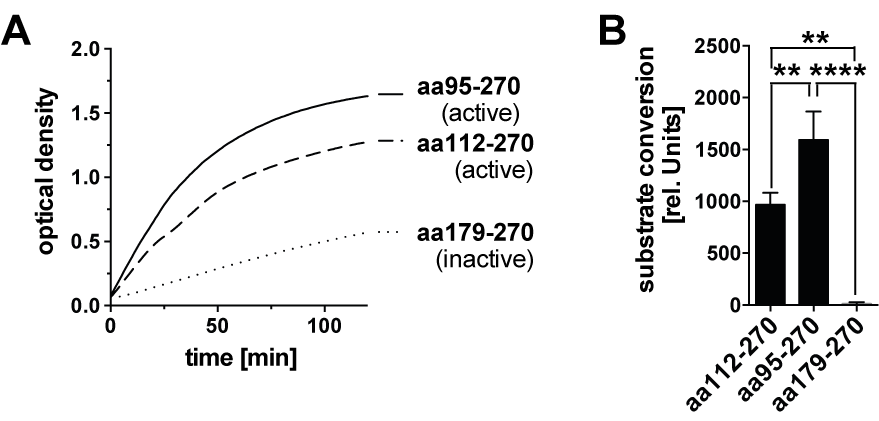


**Supplementary figure 1: Bioactive IL-33 isoforms induce NF-κB dependent substrate conversion by SEAP in HEK293-ST2L reporter cells.** HEK293-ST2L cells were treated with **(A, B)** 1 ng/ml recombinant human IL-33 isoforms aa112-270, aa95-270 and aa179-270 for 22h. **(A)** SEAP activity was assessed by photometrical measurement of the converted substrate QUANTI-Blue at 635 nm for 120 minutes. Representative data of n=3 independent experiments. **(B)** The substrate conversion in relative Units corresponds to the respective bioactivity of IL-33 aa112-270, aa95-270 and aa179-270. Data are shown as mean ± SD of n=3 independently performed experiments with ** p < 0,01 and **** p < 0,0001 using *one-way ANOVA with Tukey’s posttest*.


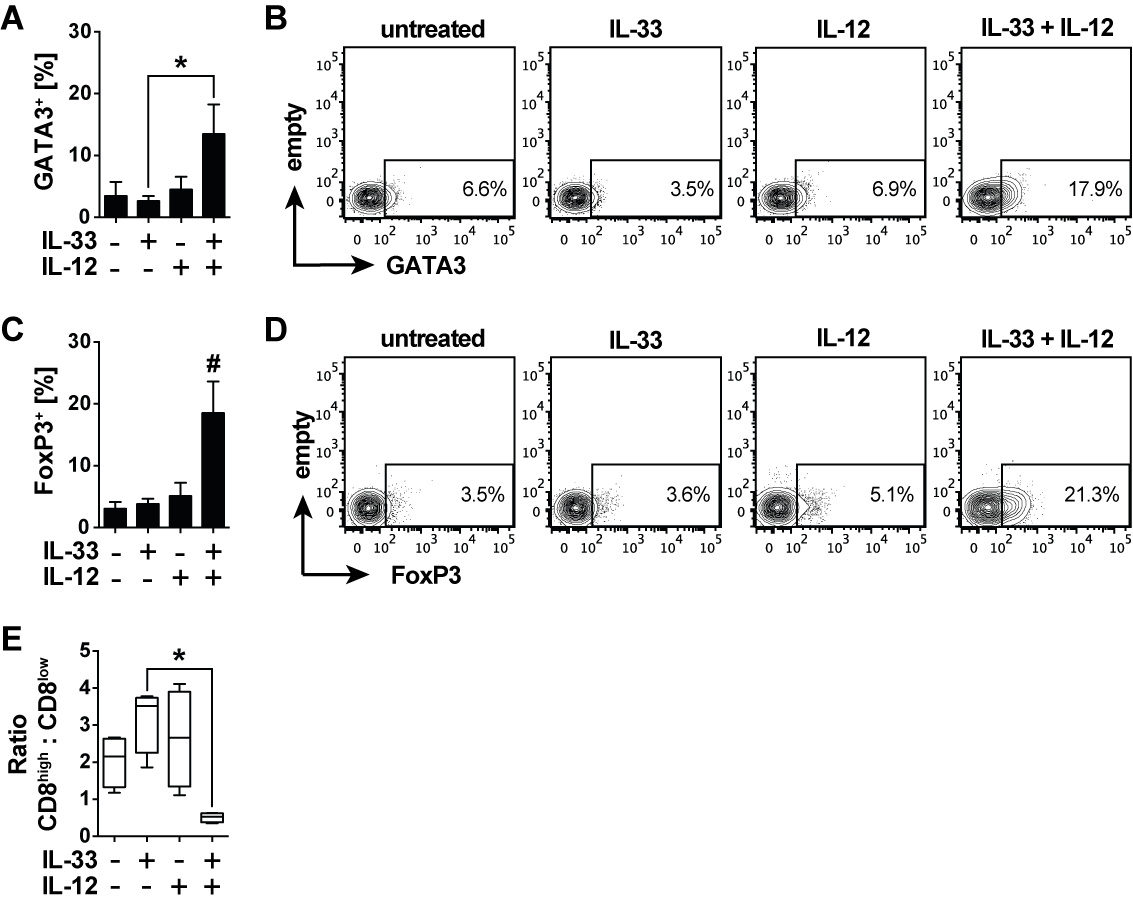


**Supplementary figure 2: IL-33 inhibits IL-12 dependent and TCR-independent effector functions by induction of GATA3 and FoxP3 during nutrient deprivation.** CD8^+^ T cells were treated with IL-33 and/ or IL-12 and analyzed by flow cytometry. **(A)** Percentage of GATA3^+^ and **(C)** FoxP3^+^ CD8^+^ T cells. Representative flow cytometry data for intracellular expression of **(B)** GATA3 and **(D)** FoxP3, respectively. **(E)** Ratio of CD8^high^ to CD8^low^ T cells. Data are shown as mean ± SD of n=4 different donors with at least n=3 independently performed experiments. */^#^ for p ≤ 0.05 using *Friedman test with Dunn’s posttest.*


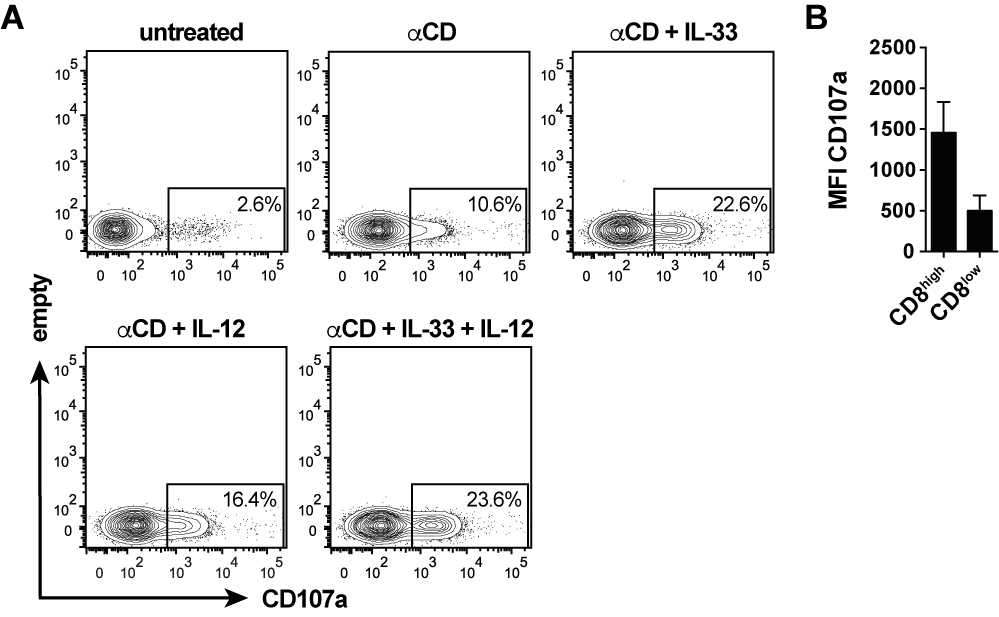


**Supplementary figure 3: TCR-, IL-12- and IL-33-dependent co-activation mediates cytotoxic degranulation of CD8^high^ T cells.** **(A)** Representative flow cytometry data of CD107a cell surface expression during stimulation of CD8^+^ T cells with αCD, IL-33 and/ or IL-12. **(B)** Mean fluorescence intensity (MFI) of indicator of cytotoxicity CD107a on the cell surface of CD8^high^ and CD8^low^ T cells upon co-stimulation of IL-33, IL-12 and the TCR. Data are shown as mean ± SD of n=4 different donors with at least n=3 independently performed experiments.
